# Analysis of Bioactive Amino Acids from Fish Hydrolysates with a New Bioinformatic Intelligent System Approach

**DOI:** 10.1038/s41598-017-10890-1

**Published:** 2017-09-07

**Authors:** Mohamed Abd Elaziz, Ahmed Monem Hemdan, AboulElla Hassanien, Diego Oliva, Shengwu Xiong

**Affiliations:** 1 School of Computer Science and Technology, Wuhan University of Technology, Wuhan, China; 2 Faculty of Veterinary Medicine, Kafrelsheikh University, Kafrelsheikh, Egypt; 3 Faculty of Computers and Information, Cairo University, Cairo, Egypt; 4 Departamento de Ciencias Computacionales, Universidad de Guadalajara, CUCEI, Av. Revolucion 1500, Guadalajara, Jal Mexico; 5 Hubei Collaborative Innovation Center of Basic Education Information Technology Services, Hubei University of Education, Wuhan, China; 6 Department of Mathematics, Faculty of Science, Zagazig University, Zagazig, Egypt

## Abstract

The current economics of the fish protein industry demand rapid, accurate and expressive prediction algorithms at every step of protein production especially with the challenge of global climate change. This help to predict and analyze functional and nutritional quality then consequently control food allergies in hyper allergic patients. As, it is quite expensive and time-consuming to know these concentrations by the lab experimental tests, especially to conduct large-scale projects. Therefore, this paper introduced a new intelligent algorithm using adaptive neuro-fuzzy inference system based on whale optimization algorithm. This algorithm is used to predict the concentration levels of bioactive amino acids in fish protein hydrolysates at different times during the year. The whale optimization algorithm is used to determine the optimal parameters in adaptive neuro-fuzzy inference system. The results of proposed algorithm are compared with others and it is indicated the higher performance of the proposed algorithm.

## Introduction

Nowadays, Peptides with their bioactive amino acids play a functional role at many pharmaceutecal and nutriceutical industries. In this trend, we need intelligent, accurate and fast bioanalytical measurements to assess the analytes of interest with variable concentrations in variable conditions. As we should promote specific production, enhance quality control processes and show food metabolic studies more clearly. Unfortunately, these industries face great technical problems with the bioactive amino acids production, the major problem is amino acid analysis in foodstuffs as they are destructed during acid hydrolysis in the preparation step, this problem can be greatest with the essential amino acids likely to be limiting in functional diets “methionine and cystine”, Lysine, threonine, and tryptophan. All amino acids have already been commercialized as nutraceuticals^1^. So, there is an urgent need to apply intelligent algorithms for not only detection but also the characterization of novel bioactive peptides in the protein^2^. Peptides from Fish protein hydrolysates differ so widely in their composition that “Lab” analytical methods would need to be more specific for each type, but these methods are time-consuming. Thus, compromises between the Lab and computerized analytical methods are often necessary, especially to promote the best utilization of great functional and nutritional benefits in protein^3^.

Fish proteins have variable but functional and biological applications^4^. They are the source of secretagogues, calciotropic hormones and growth factors^5^. Their bioactive amino acids provide important functional and biological roles such as antihypertensive, antioxidant and immune modulatory activities. They perform the regulation of the blood pressure through inhibition angiotensin converting enzyme activity. As well as the antihypertensive role, they perform antioxidant roles through scavenging activity that prevent oxidation process^6^. They also enhance the capacity of lymphocyte proliferation, percent of T-helper cells in spleen and secretion of interferon plus cytokines. So, they have a great role in clinical diet formations which used in specific diseases. In allergic patients, enzymatic protein hydrolysates and a mixture of specific amino acids have a great importance to decrease immune-mediated hypersensitive reactions^7^. Not only allergic patients but also patients with cancer and hepatic encephalopathies as they suffer from disorders in metabolism^8^.

Many studies have found that the functional properties of amino acids are related to the concentration in the diet and to the source of amino acids. As an example, fish-derived bioactive peptides are more functionally active than other sources^9^. In this paper, we estimated the concentration of bioactive amino acids in fish by-product protein hydrolysates with studying the effect of variable environmental temperature over the year. We aim to study the dynamic properties of functional amino acids as it has a great importance as it detects the functional quality of the extracted protein hydrolysates at different times^10^. This also has a vital role in pharmaceutical dynamic properties. So, we can target the produced protein hydrolysates to certain drugs based on amino acids concentration levels. As an example, Patients with the liver disease show a plasma amino acid imbalance with high levels of tyrosine and phenylalanine and low level of valine leucine and isoleucine^11^. Therefore, the new analytical algorithms help to choose the specific amino acids which has an essential role in the treatment of patients with chronic liver diseases as an example. The optimum supply of amino acids is also necessary to enhance hepatic regeneration and immunologic host defense^12^ as well as normalization of plasma amino acid profile^13^. Finally, The previous functional and bioactive properties struggle the challenge of many changes in the environmental conditions and variation in the temperature, so the optimal exploitation of bioactive amino acids for human nutrition and health possesses an exciting scientific and technological challenge while at the same time offering potential for commercially successful applications.

In this paper, we proposed an a new prediction approach based on adaptive neuro-fuzzy inference system (ANFIS)^14, 15^ to improve the performance of predicting the amino acids concentration in fish. However, determining the optimal values for the parameters of the memberships function and weights between layers of ANFIS model is the main problem in ANFIS. The gradient descent approaches are the popular algorithms that used to learn the parameters of ANFIS. However, the gradient is computed at each iteration and it can be stuck with local point and therefore not a global solution can be determined^16^. To solve these drawbacks, the meta-heuristics like genetic algorithms (GAs)^17^ and particle swarm optimization (PSO)^18, 19^ are used. However, GAs are slow convergence speed, whereas PSO is sensitive to neighborhood topology. So, the whale Optimizer (WO) algorithm is used to solve this problem^20^.WO is a new metaheuristic inspired that emulates the humpback whales^20^. In WO, there are three steps are used to mimic the hunting behavior: tracking, encircling and attacking the prey.

The main goal of this paper is to analyze the amino acid dynamics at variable temperature values with improving the performance of intelligent system (ANFIS based WO algorithm) to obtain the highest predictive importance.

## Adaptive Neuro-Fuzzy Inference System (ANFIS)

**Figure 1:**
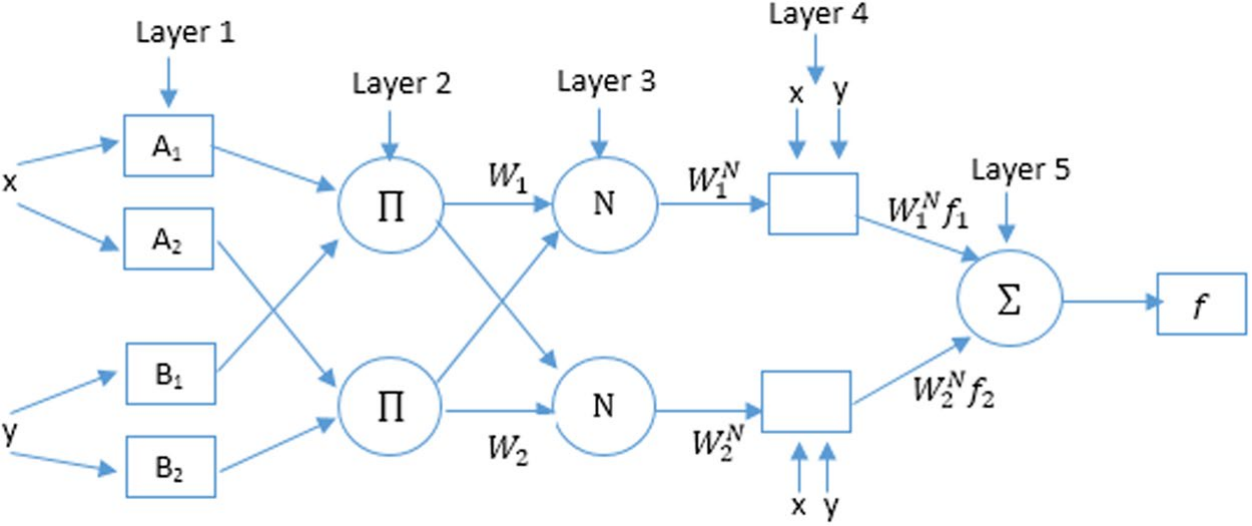
The five layers of ANFIS model.

The adaptive neuro-fuzzy inference system (ANFIS) is a hybrid of both neural network (NN) and fuzzy logic^14, 15, 21^. The structure of ANFIS is illustrated in Fig. 1, in which the ANFIS consists of five layers. The input data (*x* and *y*) are presented to each node in the first layer and the output is computed by using the generalized Gaussian membership function $\mu (x)$ as:

1 $${O}_{1i}={\mu }_{{A}_{i}}(x),i=1,2,{O}_{1i}={\mu }_{{B}_{i-2}}(y),i=3,4,\mu (x)={e}^{-{((x-{\rho }_{i})/{\sigma }_{i})}^{2}}$$

where ${A}_{i},{B}_{i}$ are the membership values of the ${\mu }_{A}$ and ${\mu }_{B}$, respectively. ${\rho }_{i}$ and ${\sigma }_{i}$ are represent the mean and standard deviation of data respectively. The output of each node in the first layer is passed to the second layer and the firing strength of a rule (${w}_{i}$) is computed as:

2 $${w}_{2i}={\mu }_{{A}_{i}}(x)\times {\mu }_{{B}_{i-2}}(y)$$

Then in third layer the normalized firing strength (${\bar{w}}_{i}$) is computed for each node as:

3 $${O}_{3i}={\bar{w}}_{i}={w}_{i}/(\sum _{i\mathrm{=1}}^{2}{w}_{i}),$$

The normalized firing strength and the function ${f}_{i}$ is passed to each node in the fourth layer (an adaptive node) and its output is computed as:

4 $${O}_{4i}={\bar{w}}_{i}{f}_{i}={\bar{w}}_{i}({p}_{i}x+{q}_{i}y+{r}_{i})$$

where ${p}_{i},{q}_{i}$ and ${r}_{i}$ is the consequent parameters of the node. In the last layer, there is a single node and it is output is defined as.

5 $${O}_{5}=\sum _{i}{\bar{w}}_{i}{f}_{i}$$

The ANFIS parameters are divided into two sets, the consequent and premise parameters. All of these parameters are needed to update in learning process until the target is achieved. There are some approaches used to learn the ANFIS parameters such as the Least Square Method (LSM) is used to find the optimal values for both sets of the parameter. However, its convergence is slow and the hybrid algorithm that combines the LSM and the backpropagation (BP) algorithm is used to solve this problem^17^. This algorithm is susceptible to get stuck at local optima. To overcome this drawback, this paper introduces a new evolutionary technique, namely, Whale algorithm as in the following section.

## The Whale Optimization Algorithm

The whale optimization (WO) algorithm is a new swarm technique that emulates the humpback whales^20^. In WO algorithm, the search starts by generating a random population of whales (solutions). These whales attacking (optimization) their prey (${\overrightarrow{X}}^{\ast }$) in either Encircling or Bubble-net method after determining the location of the prey.

In the encircling method^20^: The position of humpback whales are updated according to the best position as^20^:

6 $$\overrightarrow{D}=|\overrightarrow{C}\odot \overrightarrow{X}\ast (t)-\overrightarrow{X}(t)|$$

7 $$\overrightarrow{X}(t+\mathrm{1)}=|\overrightarrow{X}\ast (t)-\overrightarrow{A}\odot \overrightarrow{D}|$$

where $\overrightarrow{D}$ is the distance between the position of the prey ($\overrightarrow{X}{(t)}^{\ast }$) and the and other whales ($\overrightarrow{X}(t)$) at the current iteration number $t$. The two coefficient $\overrightarrow{A}$ and $\overrightarrow{C}$, and are calculated as follows:

8 $$\overrightarrow{A}=2\overrightarrow{a}\odot \overrightarrow{r}-\overrightarrow{a},\,\,\,\overrightarrow{C}=2\overrightarrow{r}$$

where $r\in \mathrm{[0},\mathrm{1]}$ is random number, and the parameter $\overrightarrow{a}$ is decreased linearly from 2 to 0 as the iteration increased.

There are two approaches to simulate the bubble-net behavior. The first approach is the shrinking encircling that achieved by using equation (8), also, $\overrightarrow{A}$ is decreased. The second approach is the spiral updating position: This method is used to simulate the helix-shaped movement of humpback whales around prey:

9 $$\overrightarrow{X}(t+\mathrm{1)=}\overrightarrow{D}\text{'}\odot {e}^{bl}\odot cos\mathrm{(2}\pi l)+{\overrightarrow{X}}^{\ast }(t)$$

where $\overrightarrow{D}\text{'}=|{\overrightarrow{X}}^{\ast }(t)-\overrightarrow{X}(t)|$ is the distance between the whale and prey, $b$ is a constant for defining the shape of the logarithmic spiral, $l$ is a random number in [−1, 1], and $\odot$ is an element-by-element multiplication. The humpback whales can simultaneously swim around the prey through a shrinking circle and along a spiral-shaped path^20^.

10 $$\overrightarrow{X}(t+\mathrm{1)}=\{\begin{array}{cc}{\overrightarrow{X}}^{\ast }(t)-\overrightarrow{A}\odot \overrightarrow{D} & if\,\,p\ge 0.5\\ \overrightarrow{D}^{\prime} \odot {e}^{bl}\odot cos\mathrm{(2}\pi l)+{\overrightarrow{X}}^{\ast }(t) & if\,\,p < 0.5\end{array}$$

where a random probability $p\in \mathrm{[0},\mathrm{1]}$ is used to switch between the spiral model or the shrinking encircling mechanism to improve the position of whales.

In exploration phase, the whales search about the prey in a random from. The position of a whale is updated by selecting a random whale rather than ${X}^{\ast }$ as follows:

11 $$\overrightarrow{D}=|\overrightarrow{C}\odot {\overrightarrow{X}}_{rand}-\overrightarrow{X}(t)|$$

12 $$\overrightarrow{X}(t+\mathrm{1)}=|{\overrightarrow{X}}_{rand}-\overrightarrow{A}\odot \overrightarrow{D}|$$

where ${\overrightarrow{X}}_{rand}$ is a random whale’s position selected from the population.

## The proposed prediction Algorithm

In this section, the proposed algorithm for predicting the bioactive amino acids concentration in fish. This algorithm is the ANFIS based on WO (called ANFIS-WO), where this approach consists of five layers. The inputs variables to the first layer are (Moisture, fat, ash, Crude protein, and Temperature) and the output of layer 5 is the amino acids concentrations.

The proposed algorithm starts by normalizing dataset then the fuzzy c mean (FCM) is used to determine the number of membership functions. The next step is to construct the ANFIS based on the number of membership function. The parameters in ANFIS are updated based on WO algorithm, that used square euclidian distance as a fitness function is defined as:

13 $$fitness\,function={\Vert out-pred\Vert }^{2}$$

where the WO algorithm is started by generating a population with a random position for each whale that represents the parameters of ANFIS. Then the fitness function for all population is computed and the global objective function is determined. The value of $a$ is decreased from 2 to 0 and for each whale in the population the $A$ and $C$ are computed based on equations (8) and (??) respectively. Then the position of current whale is updated based on the value of $p$, where if $p\,\mathrm{ > 0.5}$ then the current position becomes the best position otherwise the position is based on either equations (6–7) or equations (11–12) based on if $|A| < \,0.5$ or $|A|\ge 0.5$ respectively. The WO still update the position until the stop condition is satisfied, the best solution is passed to ANFIS.

**Figure 2:**
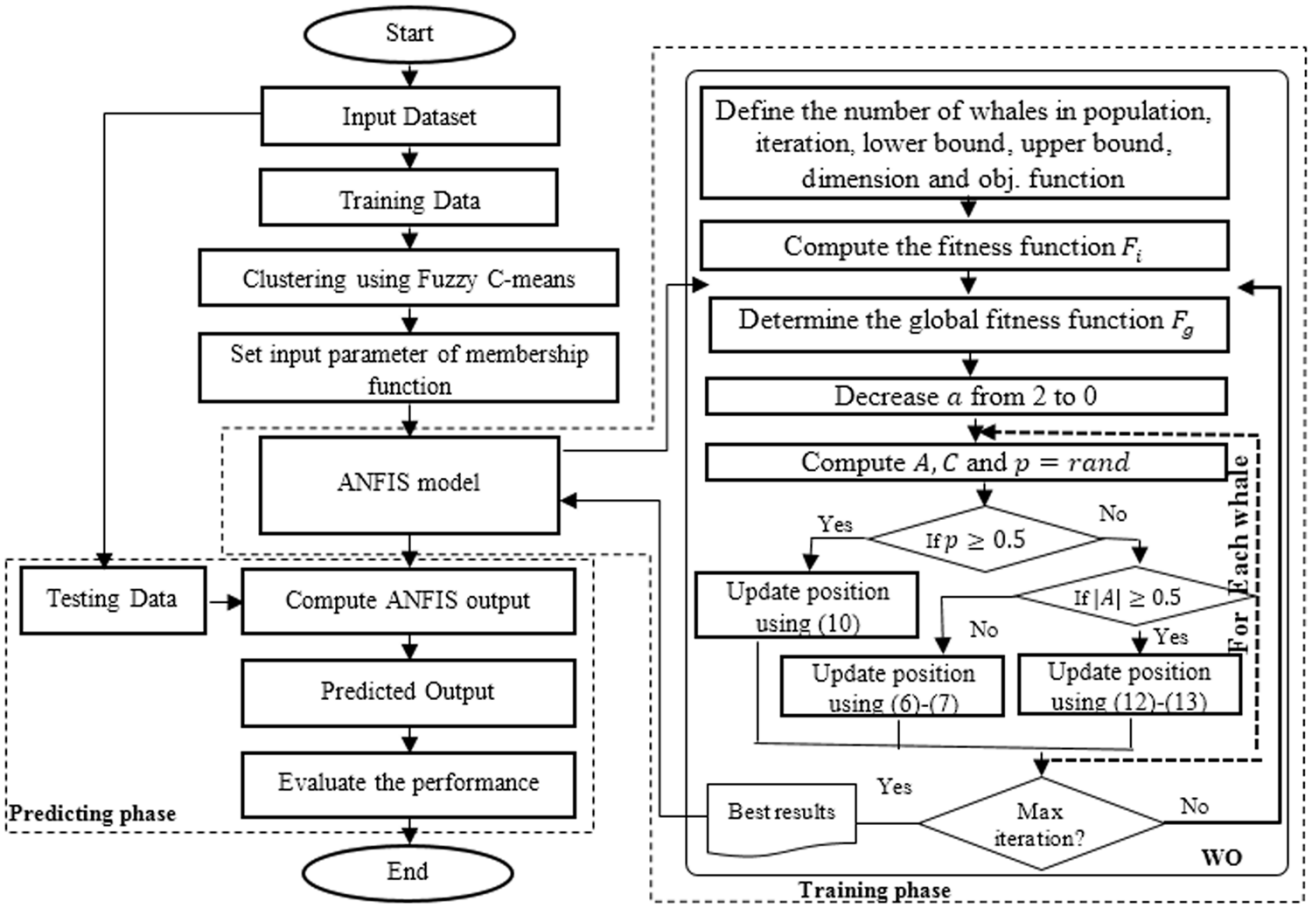
Flowchart of Proposed model.

The training phase is finished, if the stop conditions (maximum number of iteration and error less than small value) are satisfied. In the predicting phase, the test data set in introduced to the ANFIS that predict the output and the performance of the output is evaluated. The proposed algorithm is illustrated in Fig. 2.

## Experimental Results and Discussion

In the experiments, the data is divided into training and testing sets by using two methods, in the first method, the data is split randomly into 70% samples for the training set and the rest 30% as a testing set. However, the random division may be not accurate and can cause bias in the results of prediction, so, in order to avoid this limitation there are four strategies can be used as a second method. For example. the *N*-fold cross-validation test, sub-sampling test, independent dataset test and jackknife cross-validation test, in which these strategies have been widely used to examine the performance of a prediction model^22–26^. In this study, the *N* fold cross-validation test, (here 10 fold) was used to investigate the performance of the prediction model.

The ANFIS-WO algorithm was compared with five models, namely, ANFIS-PSO, ANFIS-GA, ANFIS, IBK, SMO, and SVM. The experiments were implemented in Matlab R2014b and Windows 10 (64-bit). The parameters are set as size of population is *n* = 25, the max iteration is $100$.

### Dataset collection

By-products of 120 fresh farmed tilapia (oreochromus niloticus) were collected every month over a year at Kafrelsheikh Governorate, Egypt (One of the most important areas in the production of tilapia in the world). We collected fish byproduct under measured parameters (weight, Sex, length, water quality, ration). Then, they were minced and stored at −30 °C till use. The following steps shows the Enzymatic hydrolysis reaction process for preparing the data samples.

- Thawing the stored by product over night in cold place(4 °C).
- 15% of the samples volume mixed with 50 ml phosphate buffer saline (pH 7.5).
- Pre-incubation at 60 °C for 20 minutes.
- Adding alcalase enzyme (2.5%)to initiate the enzymatic hydrolysis reaction.
- Heating in water bath (90 °C) for 15 minutes.
- Cooling in ice.
- Centrifuge the cooling mixture for twenty minutes at 10000 rpm then the hydrolysis degree was measured to the supernatant according to^27^.
- Supernatant extraction then freeze dried and characterized.

### Preparation Phase

#### Analysis of Tilapia fish by-product and its hydrolysates powder

The contents of tilapia fish by-product and its hydrolysates were measured according to AOAC method^28^, The protein content was determined using kjeldal method. Moisture percentage was estimated with drying method. In addition, Ash content was measured by muffle furnace. Within our study, the protein hydrolysates were extracted with alcalase enzyme with appropriate PH and temperature^29^.

#### Amino acid sequence analysis

According to^30^, stacking and separating gel were prepared using gel buffer with percentage 4% and 16% respectively. Heating the sample mixture with the buffer till 90 °C for 10 min, then loading into specific wells. Protein standards (1.06 kDa to 26.6 kDa) were also performed on the gels. Fixing, staining and destaining solutions were mixed with gel, after electrophoresis then comparing the resulted protein bands with the standard ones^31^.

### Tricine SDS-PAGE analysis

According to^30^, we performed Tricine-SDS-PAGE by preparing gel buffer with 4% and 16% stacking and separating gel respectively, then fixing solution was added to gels. After that staining solution was added before the destaining solution. Comparing the resulted bands with standard protein bands.

### Evaluation criteria

The performance and efficiency of the ANFIS-WO model is evaluated by three statistical methods, namely, Average Absolute Percent Relative Error (AAPRE)and Root Mean Square Error (RMSE) as in Table 1: where ${x}_{i}$ is the $i$-th predicted element, ${y}_{i}$ is the $i$-th measured element, and $N$ is the number of samples. ${y}_{i}$ is the average of the corresponding predicted value.

**Table 1:** Measure the performance of algorithms.

Measure	Description	Rule
Average Absolute Percent Relative Error (*AAPRE*)	measures the relative absolute deviation from the experiment output	$AAPRE=\frac{100}{N}{\sum }_{i=1}^{N}|\frac{({x}_{i}-{y}_{i})}{{y}_{i}}|$
Root Mean Square Error (*RMSE*)	Measure the differences between the predicted values and the actual values	$RMSE=\sqrt{\frac{1}{N}{\sum }_{i=1}^{N}{({x}_{i}-{y}_{i})}^{2}}$

## Results

The results of the proposed model compared with other models according to divided the data randomly are introduced in Table 2 and Figures S1–S15 in Supplementary Material which are the average of 10 runs. Where Figure S1 is the average of the algorithm overall the concentration, and from this figure we can conclude that, in general, the proposed algorithm has the best values of RMSE and AAPRE which are 1.70 and 8.23 respectively. Also, its accuracy is higher than all other versions ANFIS model that have the values 8.81, 6.35 and 8.069 for ANFIS, ANFIS-GA, and ANFIS-PSO, respectively. Also, when compared the proposed algorithm with SMO, SVM, IBK, and RF, it also still has the best solution in term of all measures. Figures S2 and S15 which indicate that The ANFIS-WO output values are nearest to the target data (not testing target only).

**Table 2:** Comparison between algorithms based on RMSE and AAPRE using random division dataset for training and testing

		ANFIS	ANFIS GA	ANFIS PSO	SMO	ANFIS WO	SVM	IBK	RF
aspartic acid	*AAPRE*	51.09	45.73	38.85	7.49	6.72	10.30	29.50	6.95
	*RMSE*	18.29	16.36	13.69	3.44	2.44	4.29	10.95	2.80
glutamic acid	*AAPRE*	36.79	33.81	27.32	30.96	7.38	14.78	17.84	8.52
	*RMSE*	23.00	20.82	16.86	21.60	5.08	11.21	11.05	5.66
serine	*AAPRE*	44.17	38.26	33.86	35.38	12.82	22.40	21.92	13.22
	*RMSE*	8.52	7.45	6.84	7.51	2.56	5.27	4.16	2.77
glycine	*AAPRE*	30.51	28.27	54.82	31.93	1.84	25.99	19.01	1.87
	*RMSE*	16.99	15.81	33.73	19.26	1.03	17.25	11.12	1.74
alanine	*AAPRE*	15.75	13.43	13.71	4.74	0.46	5.57	8.15	0.47
	*RMSE*	6.88	5.75	5.92	2.23	0.23	2.47	3.80	0.27
cysteine	*AAPRE*	97.99	36.82	76.95	422.29	30.27	290.34	58.51	31.49
	*RMSE*	0.56	0.22	0.44	2.85	0.19	2.00	0.34	0.20
tyrosine	*AAPRE*	23.01	8.41	15.80	22.99	3.98	14.19	14.13	5.14
	*RMSE*	2.85	1.12	1.98	3.35	0.58	2.40	1.75	0.68
Arginine	*AAPRE*	22.13	24.44	11.84	13.03	7.26	7.20	13.47	8.86
	*RMSE*	5.69	6.31	3.31	3.60	1.99	2.17	3.50	1.97
proline	*AAPRE*	41.43	33.42	34.57	3.92	7.16	11.37	21.86	7.81
	*RMSE*	13.06	10.72	10.84	1.52	2.64	4.13	6.89	2.69
valine	*AAPRE*	13.81	12.81	15.59	4.49	11.06	5.38	11.87	8.9781
	*RMSE*	2.95	2.75	3.31	1.06	2.50	1.26	2.54	2.75
Methionine	*AAPRE*	27.76	25.66	24.97	7.24	14.82	4.53	16.39	13.61
	*RMSE*	3.57	3.29	3.21	1.19	2.01	0.72	2.10	1.84
Isoleucine	*AAPRE*	33.64	17.11	35.57	37.86	4.49	24.29	17.80	4.61
	*RMSW*	5.25	2.84	6.42	7.16	0.94	5.08	2.88	0.98
leucine	*AAPRE*	31.28	19.68	14.32	5.88	2.89	5.39	12.23	3.31
	*RMSE*	10.59	6.60	4.83	2.26	1.31	2.02	4.18	1.46
Histidine	*AAPRE*	76.47	39.22	18.36	8.26	4.15	11.42	40.57	4.75
	*RMSE*	5.23	2.74	1.29	0.65	0.32	1.01	2.86	0.35

Moreover, the comparison results between the proposed method and the other methods according to the 10fold cross-validation are given in Table 3 and Figures S16–S30 in Supplementary Material. From these results, it can be seen that the high performance of the proposed algorithm has the better average overall concentrations, nearly, 1 and 5.64 for RMSE and AAPRE, respectively. As well as, the RF algorithm, is in the second rank which has better results than the other followed by the SVM algorithm; while the worst results are achieved by traditional ANFIS.

## Discussion

Preliminary studies were carried out in order to determine the concentration of bioactive amino acids (Figures S1 and S16) in crude protein by-products and protein hydrolysates by-products by alcalase enzyme hydrolysis in tilapia fish. Obviously, the main criteria for selection of the alcalase enzyme are its ability for high extraction of the target analytes (Figures S2 and S17). The characterization of the molecular weights of Protein hydrolysates by SDS-PAGE showed the presence of strong bands ranging between 3.5–26.7 kDa, which indicated that alcalase enzyme was able to produce small-sized peptides in 120 min. Our study shows the alcalase enzyme ability to produce low molecular weight peptides through a high degree of hydrolysis. Fish protein hydrolysates with high functional values must be rich in low molecular weight peptides, and the effective production of such peptides from Tilapia By-product indicated its potential application in functional food products^32^ Based on this, the measured proximate compositions of Tilapia by-product and Tilapia protein hydrolysates with special concern to environmental temperature effect were selected and tested.

The experimental results showed the significant effect of environmental temperature and proximate compositions on the concentration of amino acids as. According to^33^, crude protein as a proximate composition has the greatest effect on amino acid concentrations and this matched respectively with water temperature values as external factor, Fat, moisture and ash which have a great effect on the metabolism and gene expression of amino acids in fish, specially adapted to different thermal conditions Under these controlled conditions, amino acids biosynthesis represented by their concentrations can be predicted by different algorithms.

Development of ANFIS via WO algorithm shows better performance in the concentrations standard deviation of the differences between predicted and observed amino acids concentrations than showing incorrect amino acids quantity is from the true values within the biosynthesis process of whole amino acids at different temperature values. Figures S2 and S11 (and Figures S17–S26) show the best biosynthesis process expressed by aspartic and glutamic amino acids concentrations obtained within the 27–29 °C with a marked decrease at 35 °C and 38 C. It is noted that these two amino acids have the same carboxylic acid on its side chain that gives it acidic (proton-donating) and functional properties. It is noted that Aspartate can be converted into methionine and threonine that, also, gives rise to isoleucine. Although these amino acids contain different mechanisms for their regulation and concentration, ANFIS-WO algorithm gives errors with larger absolute concentration values more weight than errors with smaller absolute concentration values with the same effect of temperature for aspartic acid and isoleucine (Figures S2 and S9, and Figures S17 and S24), otherwise, methionine which found SVM the best to show its predicted values (Table 2).

According to^34^, Alanine and Valine are produced by the transamination of pyruvate molecules as given in Figure S3 (Figure S18) and Figure S4 (Figure S19), respectively. These figures show that the lowest concentrations were in between the 12–15 C; as cold temperature may affect the glycolysis process and decrease the pyruvate production which is the precursor for the alanine and valine. Because leucine is synthesized by a diversion from the valine synthetic pathway, the feedback inhibition of valine on its pathway also can inhibit the synthesis of leucine.

This biosynthesis process of the previous nonpolar amino acids alanine, as well as leucine diversion from the valine synthetic pathway, were optimized based on their concentrations with higher performance by ANFIS-WO algorithm under variable temperature measurements as shown in Figure S8 (Figure S23), but valine concentrations were predicted with the highest standard metric values to be at the highest performance with SMO Tables 2–3. Likewise, the proline amino acid (non-polar amino acid) was predicted in the best performance value with SMO as in Table 2, however, based on the results in Table 3, the SMO algorithm is in the third rank after the proposed ANFIS-WO algorithm and RF algorithm.

**Table 3:** Comparison between algorithms based on RMSE and AAPRE using 10fold cross validation.

		ANFIS	ANFIS GA	ANFIS PSO	SMO	ANFIS WO	SVM	IBK	RF
aspartic acid	*AAPRE*	28.60	15.77	17.92	4.04	4.33	4.26	7.99	3.72
	*RMSE*	24.27	7.12	9.38	1.89	1.94	1.87	3.49	7.82
glutamic acid	*AAPRE*	22.27	12.71	13.65	5.03	2.09	5.18	6.96	2.39
	*RMSE*	32.71	11.38	12.07	3.95	1.55	4.07	5.35	4.62
serine	*AAPRE*	20.76	14.44	14.43	8.50	2.85	7.77	7.06	3.79
	*RMSE*	9.36	3.77	3.86	2.07	0.71	1.84	1.67	2.40
glycine	*AAPRE*	23.09	15.19	14.99	7.95	3.21	7.03	9.20	4.18
	*RMSE*	29.20	12.65	12.39	5.97	2.35	5.12	7.17	4.63
alanine	*AAPRE*	10.32	12.41	6.93	1.78	1.91	1.94	3.15	1.52
	*RMSE*	9.13	9.15	3.86	0.92	0.94	0.98	1.58	2.95
cysteine	*AAPRE*	67.00	48.32	45.84	62.05	40.41	88.54	40.25	23.42
	*RMSE*	1.47	0.93	0.92	0.84	0.69	1.15	0.75	1.60
tyrosine	*AAPRE*	18.35	9.29	9.98	6.43	2.78	7.36	5.82	3.00
	*RMSE*	5.39	1.69	1.71	0.99	0.43	1.09	0.94	0.72
Arginine	*AAPRE*	15.07	11.21	10.90	7.08	2.39	7.27	9.79	4.49
	*RMSE*	7.96	4.09	3.99	2.38	0.79	2.35	2.88	2.00
proline	*AAPRE*	26.31	15.41	16.09	5.89	4.35	6.30	8.96	8.32
	*RMSE*	19.68	9.47	7.34	2.33	1.76	2.54	3.53	2.62
valine	*AAPRE*	7.71	4.66	4.64	1.41	1.44	1.43	2.91	2.62
	*RMSE*	3.80	1.40	1.40	0.40	0.37	0.40	0.75	4.11
Methionine	*AAPRE*	14.60	10.14	10.29	7.00	3.13	6.20	7.57	4.86
	*RMSE*	4.15	1.88	1.91	1.19	0.55	1.05	1.21	1.37
Isoleucine	*AAPRE*	22.12	13.46	13.24	9.66	1.88	8.36	7.06	3.73
	*RMSE*	8.14	3.08	2.97	1.94	0.40	1.64	1.58	2.54
leucine	*AAPRE*	15.52	9.31	9.75	3.77	2.13	3.50	6.52	3.37
	*RMSE*	12.03	4.85	4.91	1.60	0.96	1.42	2.69	4.49
Histidine	*AAPRE*	47.81	25.34	27.88	8.11	6.19	9.94	13.96	6.74
	*RMSE*	7.83	2.86	3.13	0.71	0.59	0.84	1.34	1.22

From the previous results, it can be concluded that the prediction results, nearly, for all algorithms based on the 10fold cross-validation are better than the prediction through dividing the data randomly. Also, by comparing the results of the proposed algorithm overall target data (label) that given in Figures S17–S30 with previous Figures S2–S15, it can notice the high performance in Figures S17–S30; which indicates the high efficiency of the 10fold cross-validation. Moreover, the proposed ANFIS-WO algorithm is the better over the two methods (randomly and 10fold cross-validation) of constructing the training and testing sets.

It is noted that ANFIS-WO gives the best performance predicted values for Phosphoryl creates group concentrations represented in Serine-glycine and Cysteine, Serine is the first amino acid in this family to be produced; it is then modified to produce both glycine and cysteine. In Figures S6–S10 (Figures S21–S25), the proposed algorithm shows the clear variation in the serine and glycine as well as tyrosine (Figure S4 and Figure S19) (as polar amino acids) concentrations at different times over the year which matched with the actual higher concentration values at 27–29 °C and lower concentration values at 12° C, but in Figures S12 and S27, although cysteine biosynthesis derived from serine amino acids, their concentration variation is not clear as serine. This may be due to down regulation of genes required for the synthesis of cysteine which is coded on the cys regulon. Cys regulon can actually down regulate its own transcription by binding to its own DNA sequence and blocking the RNA polymerase. In this case, N-acetyl-serine which is an effective inducer of this regulon act to disallow the binding of regulon to its own DNA sequence^35^.

In Figures SS13–SS15 and SS28–SS30, ANFIS-WO present the best-predicted value performance for basic amino acids (arginine and histidine) which possesses similar chemical property. Biosynthesis prediction of these amino acids via their concentration is so vital to give clear understand the biological dynamics of amino acids in the fish and consequently their products including protein hydrolysates.

From all previous figures we can conclude that the standard deviation, MSE and RMSE has small values for all amino- acids concentration, where the predictions are the closer to the actual data.

## Conclusion and Future work

The Prediction algorithms enhance the practical properties of fish products that have an extraordinary therapeutic and industrial roles throughout our life. In this way, we assessed the concentration levels of bioactive amino acids in protein hydrolysates extracted biotechnologically from tilapia fish product with every settled parameter aside from the water temperature, planning to optimize their concentration and their interactions with each other. In addition, it is entirely costly and time-consuming to know these concentrations by the real experimental tests, especially to conduct a large-scale project. In this paper, we have introduced a new intelligent algorithm for predict the amino acids concentration. The proposed algorithm is the ANFIS based whale optimization algorithm, in which the whale is used to improve the performance of ANFIS. The results indicate that the higher performance of ANFIS-WO algorithm when compared with other algorithms in terms of RMSE and AAPRE.

For future work, further investigations are required to identify the behavior of the proposed algorithm in different applications such as water quality and other food applications. According to the potential of the proposed method, it can be applied to, other related problems such as DNA-binding protein prediction^36^, detection of tubule boundary^37^, methylation site prediction^37, 38^, phosphorylation site prediction^39^, and protein-protein interaction prediction^40, 41^. Moreover, since user-friendly and publicly accessible web-servers represent the future direction for developing practically more useful models^42–46^, we shall make efforts in our future work to provide a web-server for the method presented in this paper.

## Electronic supplementary material

Analysis of bioctave amino acids from fish hydrolysate with a new bioinformatic intelligent system approach

Dataset
