# Supplementary material for: Analysis of Bioactive Amino Acids from Fish Hydrolysates with a New Bioinformatic Intelligent System Approach
Source: Sci Rep. 2017 Sep 7;7:10860. doi: 10.1038/s41598-017-10890-1 (PMC5589738; doi:10.1038/s41598-017-10890-1)
Supplement: Supplementary file 1 — Analysis of bioctave amino acids from fish hydrolysate with a new bioinformatic intelligent system approach [file 41598_2017_10890_MOESM1_ESM.pdf]

ANALYSIS OF BIOACTIVE AMINO ACIDS FROM FISH HYDROLYSATES WITH A NEW BIOINFORMATIC INTELLIGENT SYSTEM APPROACH

Mohamed Abd El Aziz [1,5*], Ahmed Monem Hemdan[2] , Aboul Ella Hassanien [3], Diego Oliva [4], Shengwu Xiong [1*]

[1] School of Computer Science and Technology, Wuhan University of Technology, Wuhan, China.
[2] Faculty of Veterinary Medicine, Kafrelsheikh University, Egypt.
[3] Faculty of Computers and Information, Cairo University, Cairo, Egypt.
[4]Departamento de Ciencias Computacionales, Universidad de Guadalajara, CUCEI, Av. Revolucion 1500, Guadalajara, Jal, Mexico. [5] Department of Mathematics, Faculty of Science, Zagazig University, Zagazig, Egypt,

[*] To whom correspondence should be addressed; E-mail: abd_el_aziz_m@yahoo.com, xiongsw@whut.edu.cn

This PDF file includes:
(1) List of Figures.

1. List of Figures

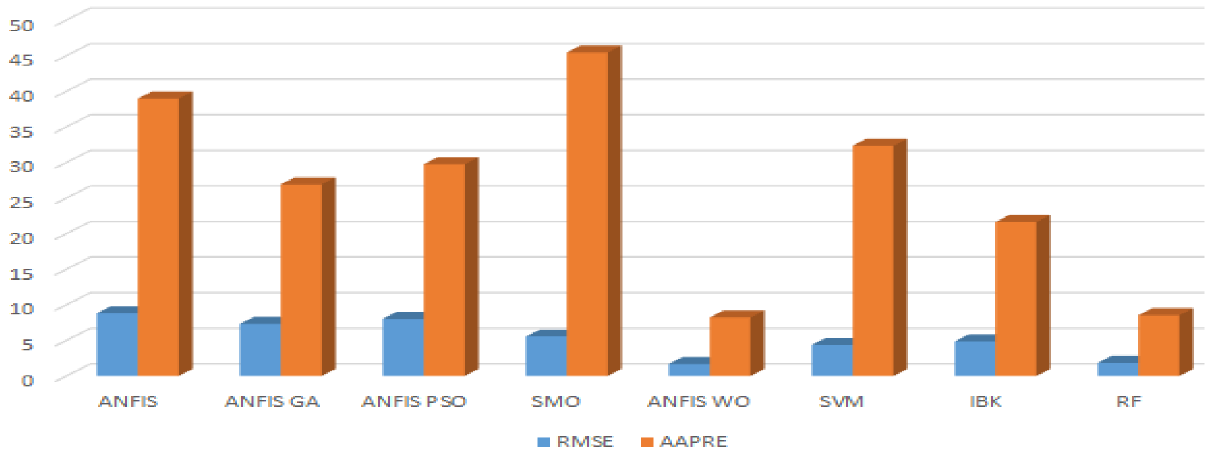

Figure S1. Average of RMSE and AAPRE of algorithms over all Concentration.

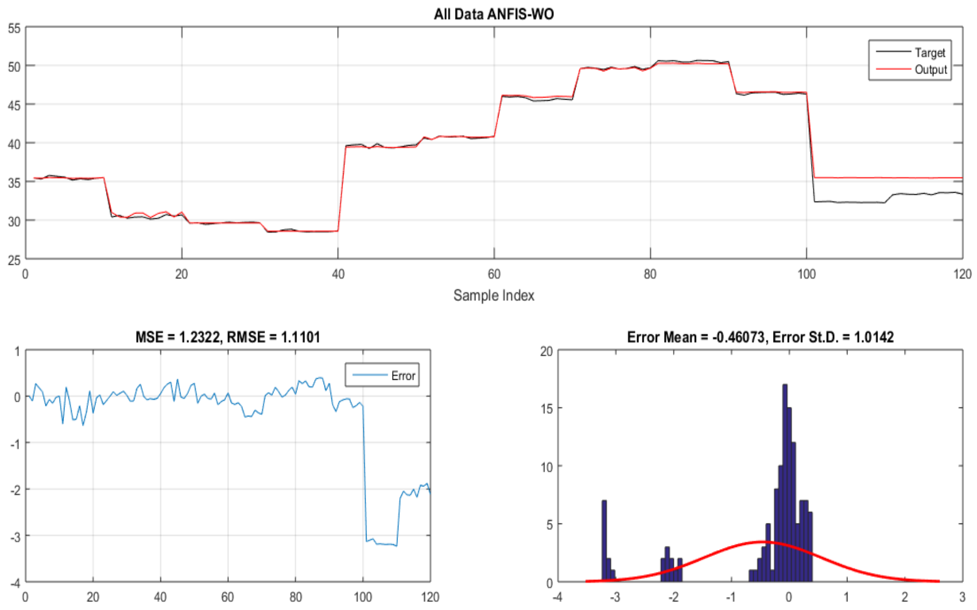

FIGURE S2. The target and predicted values of Aspartic

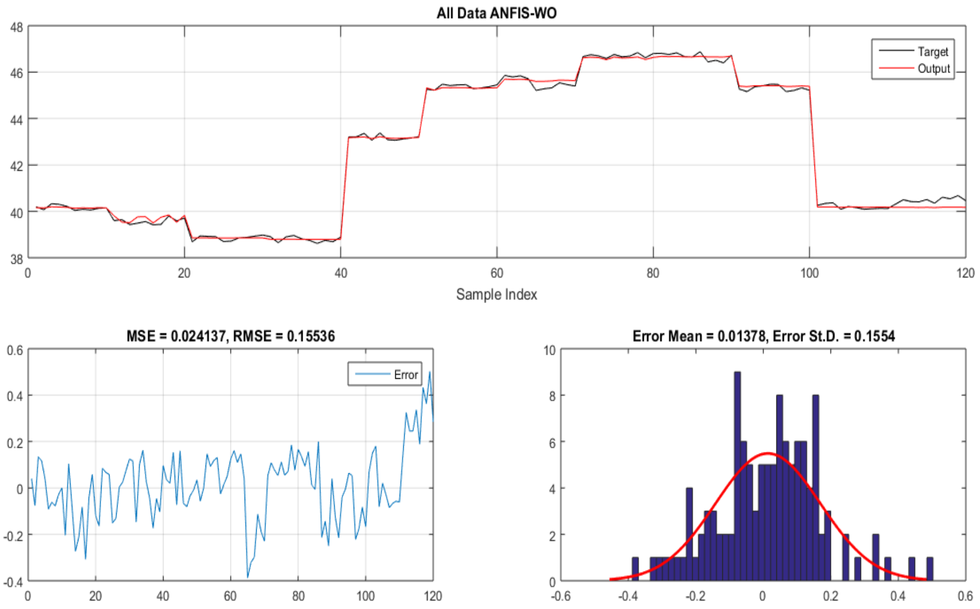

FIGURE S3. The target and predicted values of Alanine

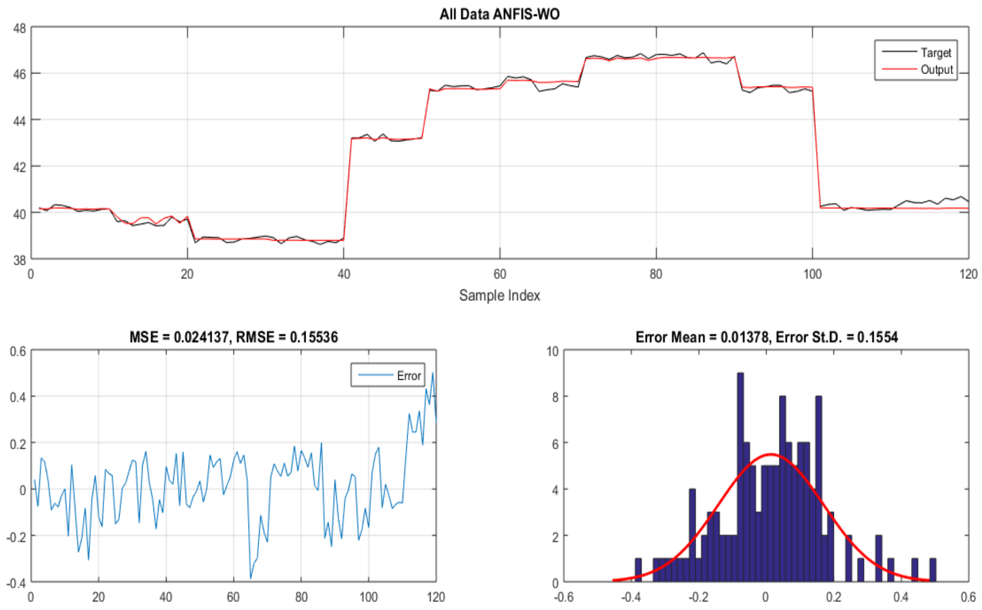

FIGURE S4. The target and predicted values of Valine

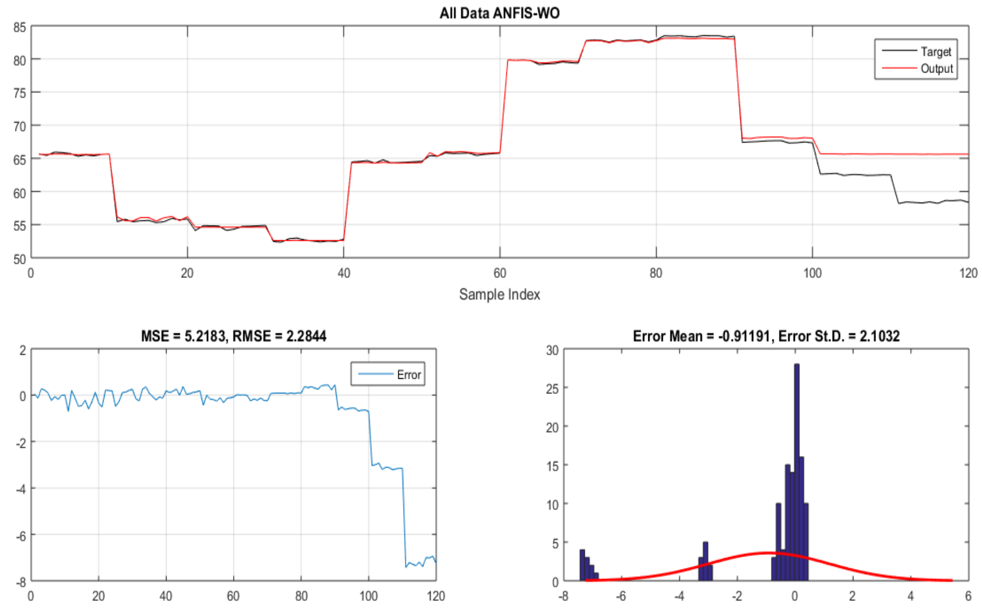

FIGURE S5. The target and predicted values of tyrosine

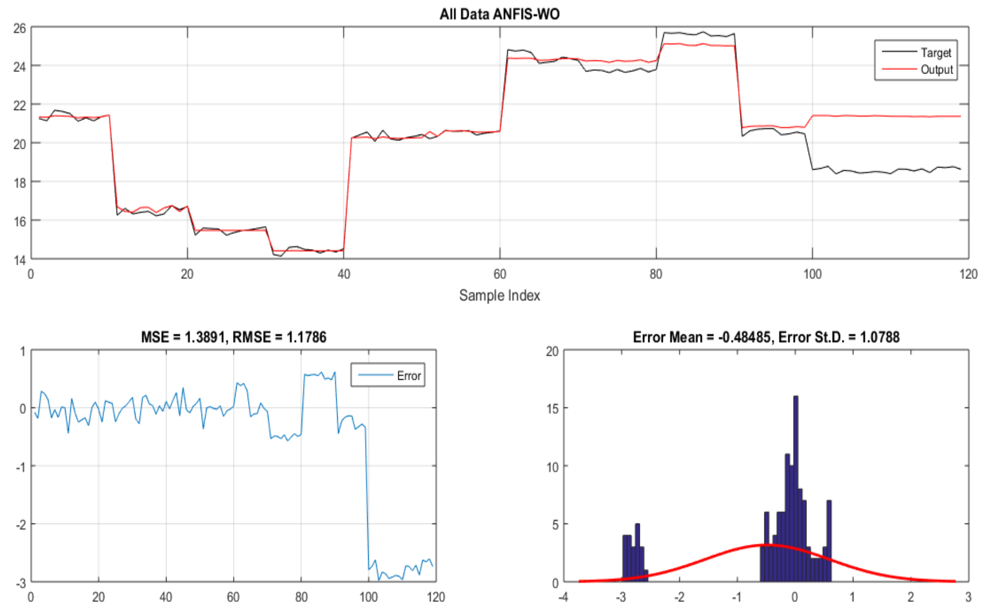

Figure S6. The target and predicted values of Serine

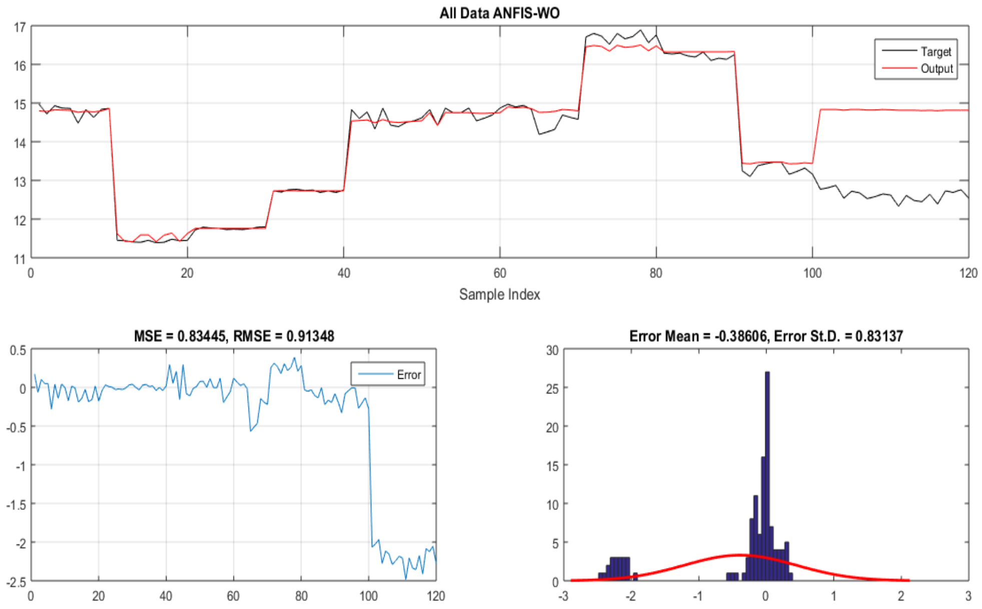

Figure S7. The target and predicted values of Methionine

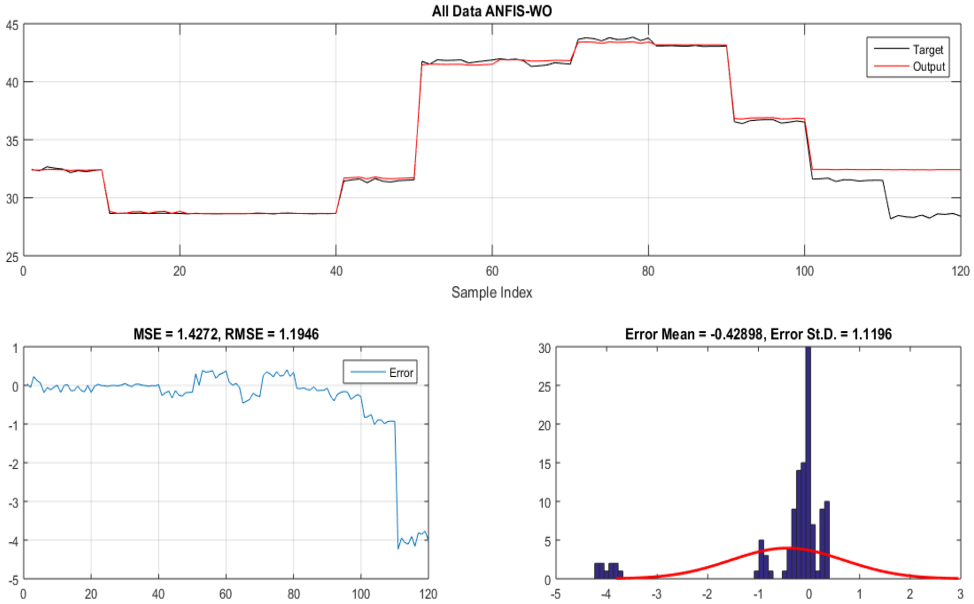

FIGURE S8. The target and predicted values of Proline

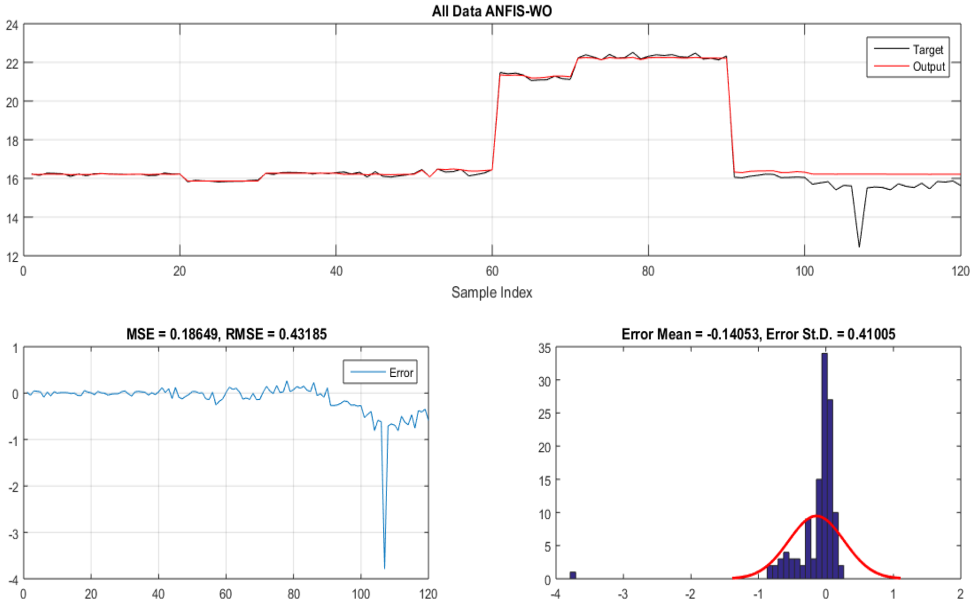

FIGURE S9. The target and predicted values of Isoleucine

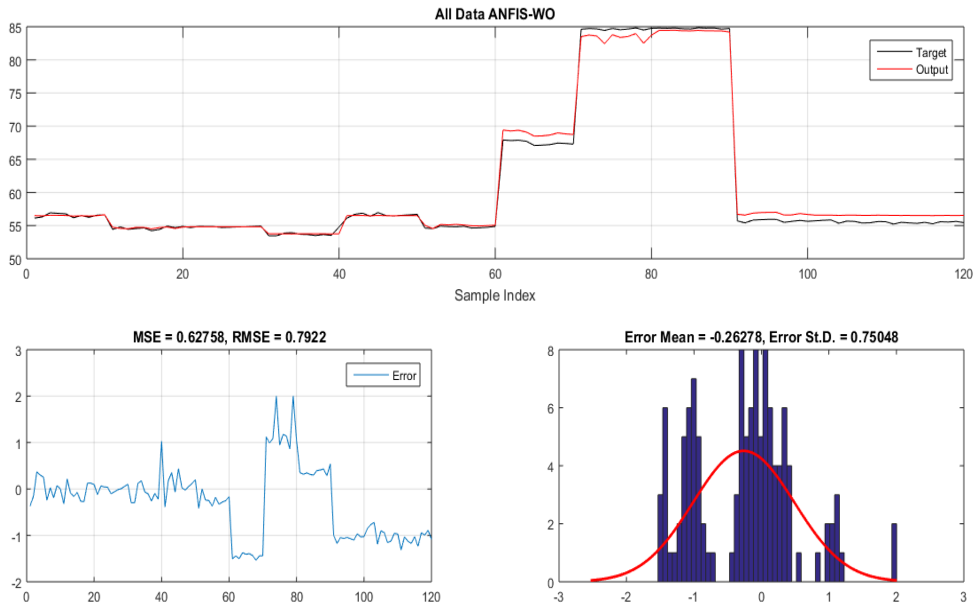

FIGURE S10. The target and predicted values of Glycine

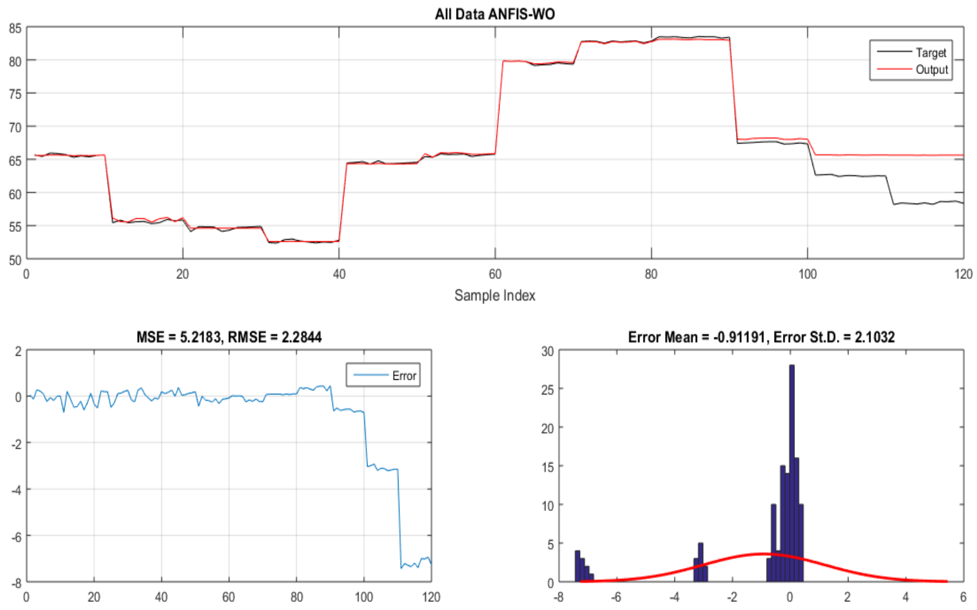

FIGURE S11. The target and predicted values of Glutamic

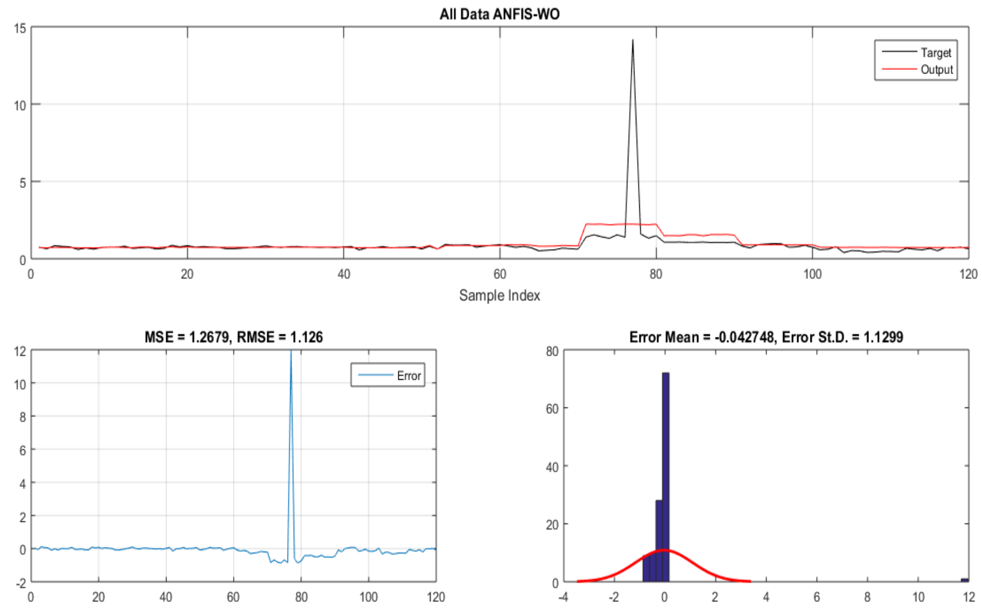

FIGURE S12. The target and predicted values of Cysteine

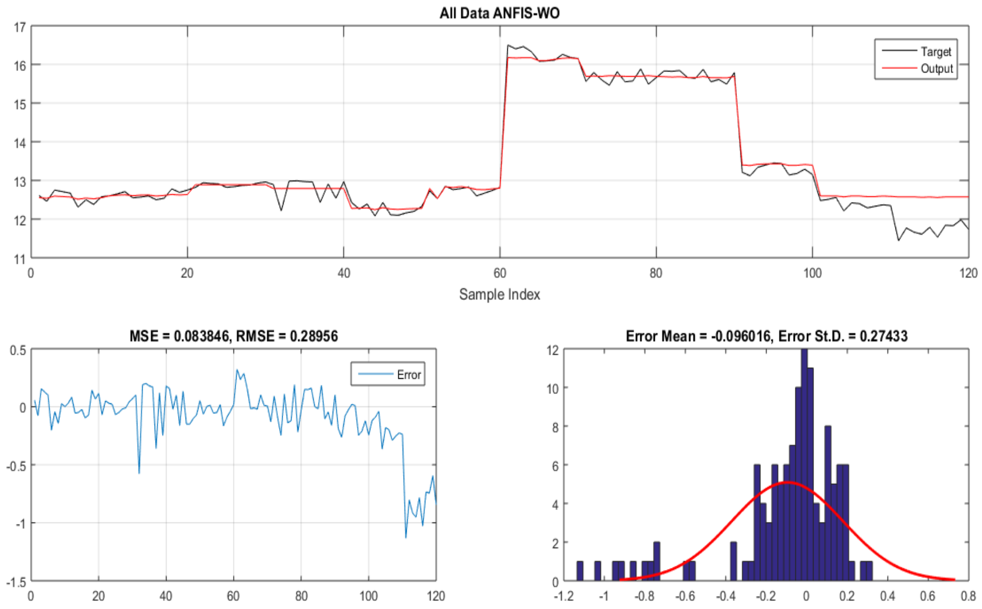

FIGURE S13. The target and predicted values of Arginine

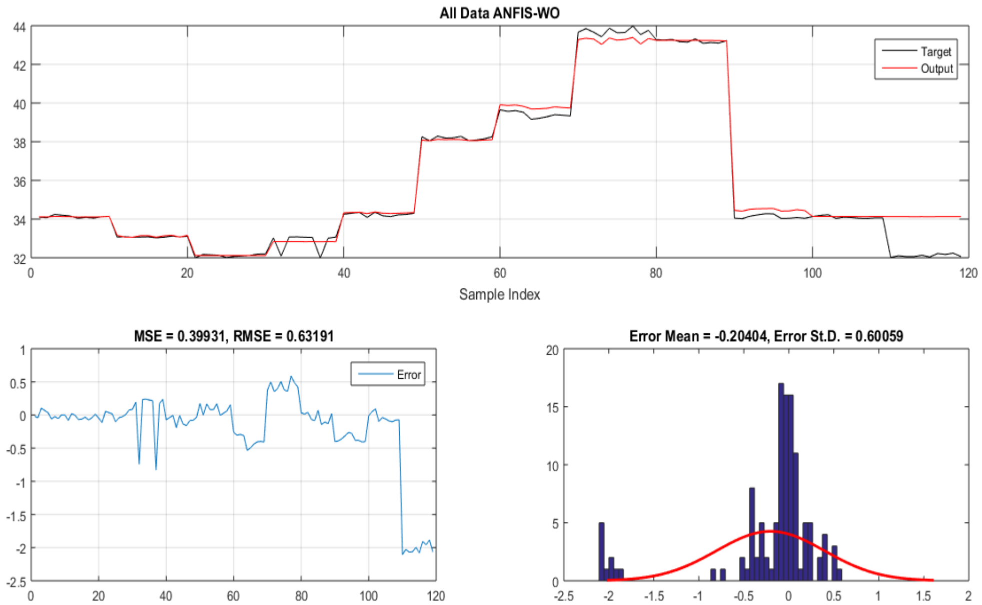

Figure S14. The target and predicted values of Leucine

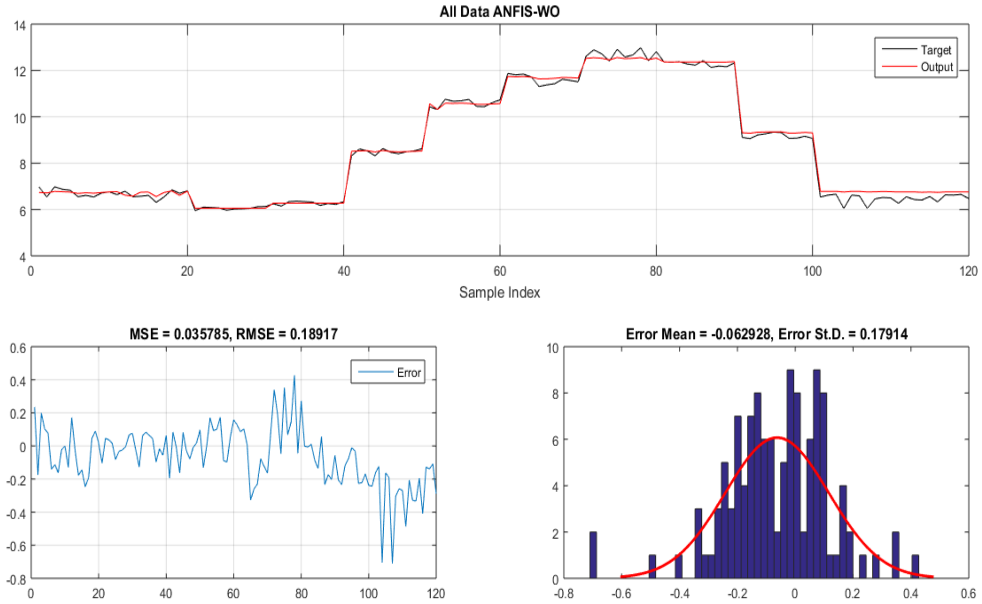

Figure S15. The target and predicted values of Histidine

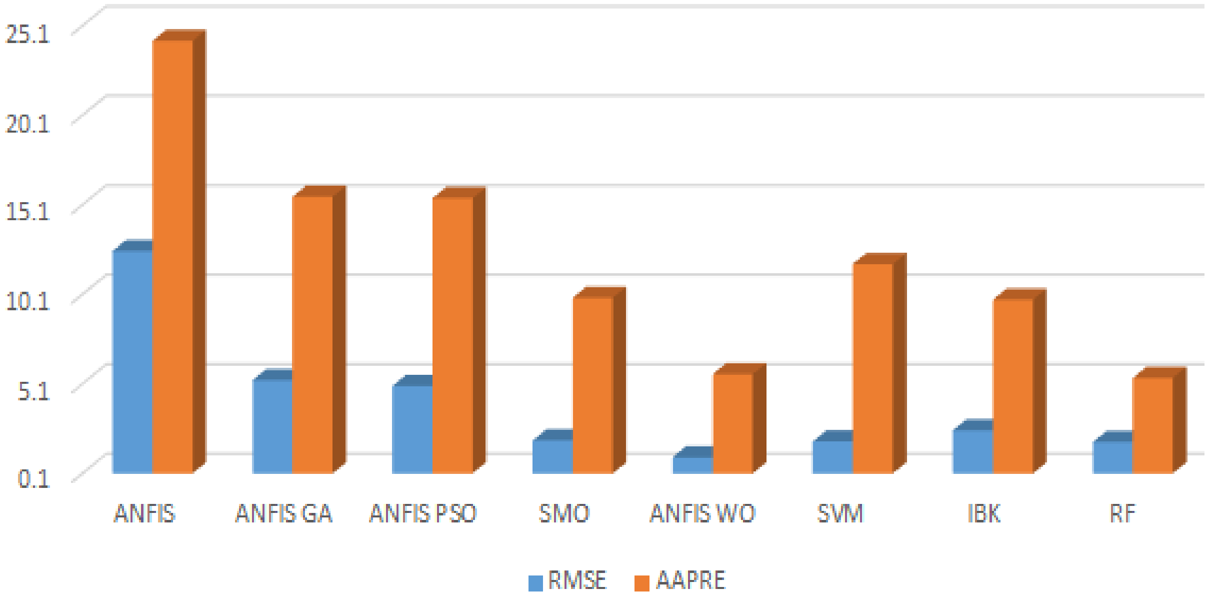

Figure S16. Average of RMSE and AAPRE of algorithms over all Concentration using 10fold cross-validation.

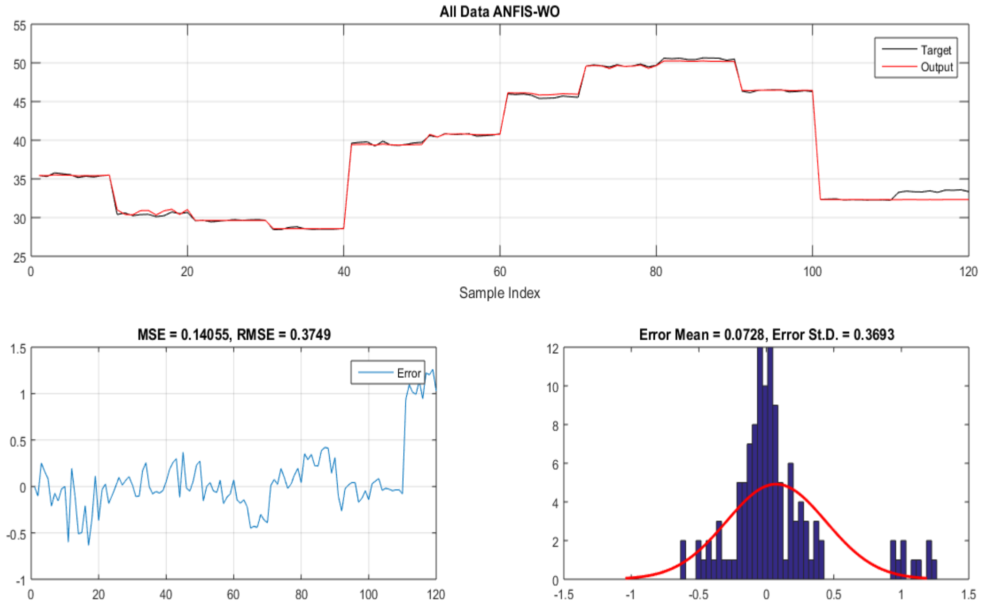

Figure S17. The target and predicted values of Aspartic using 10fold cross-validation.

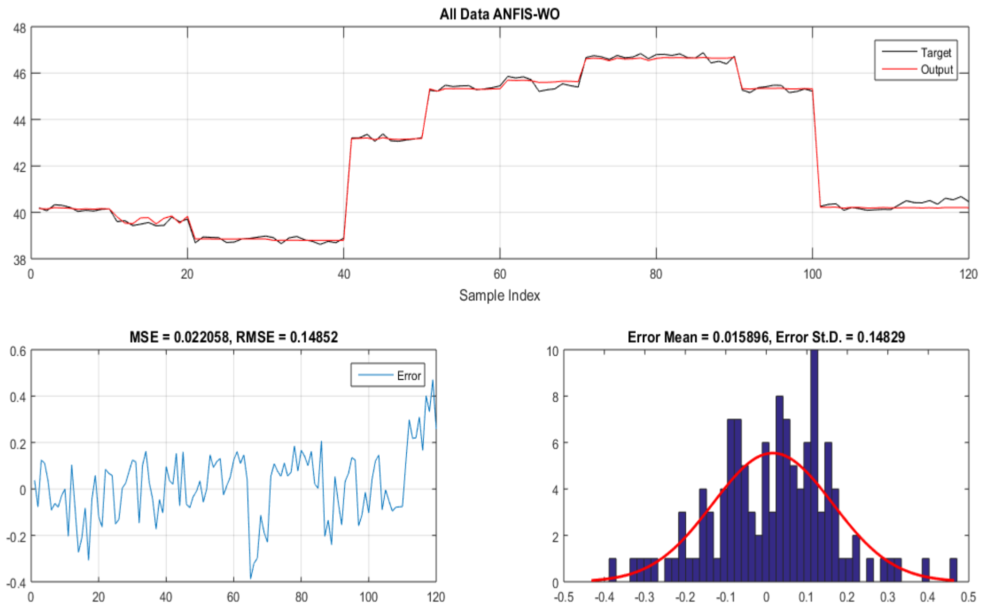

FIGURE S18. The target and predicted values of Alanine using 10fold cross-validation.

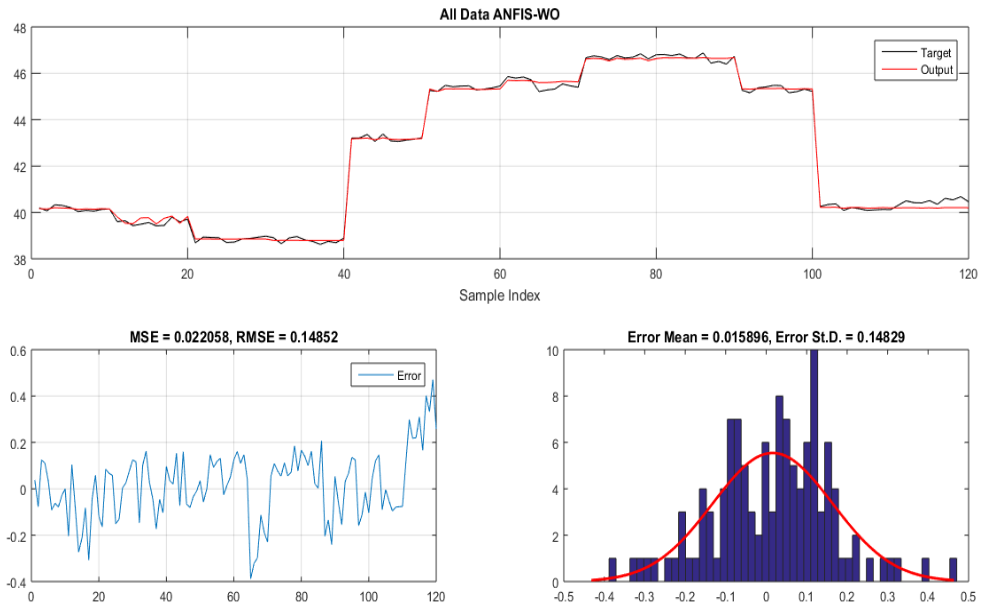

FIGURE S19. The target and predicted values of Valine using 10fold cross-validation.

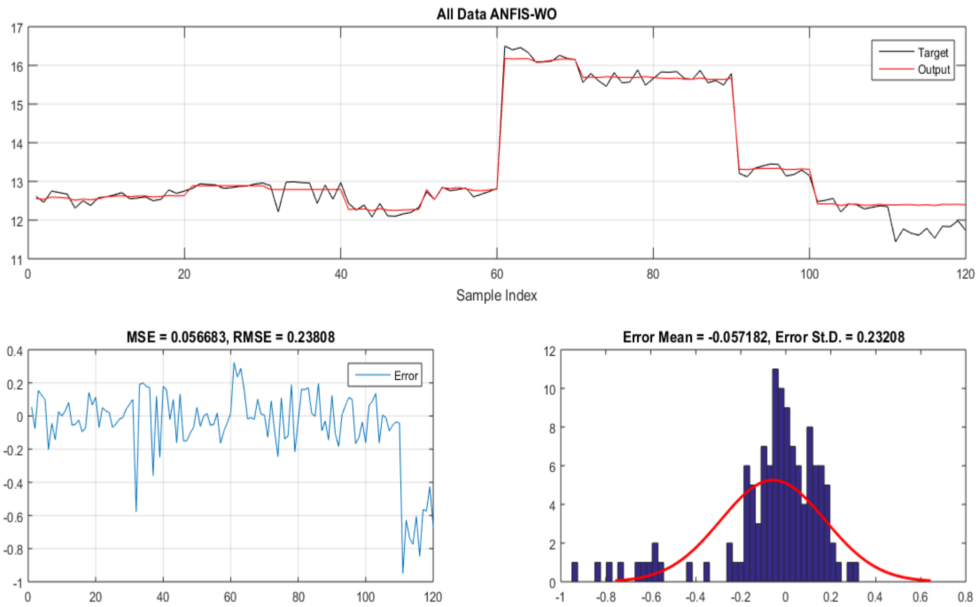

FIGURE S20. The target and predicted values of tyrosine using 10fold cross-validation.

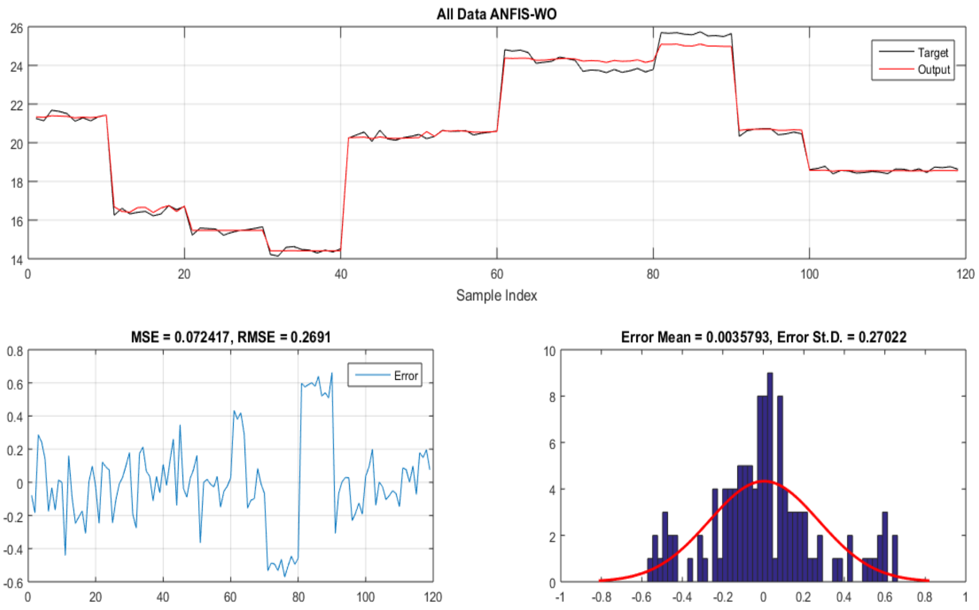

Figure S21. The target and predicted values of Serine using 10fold cross-validation.

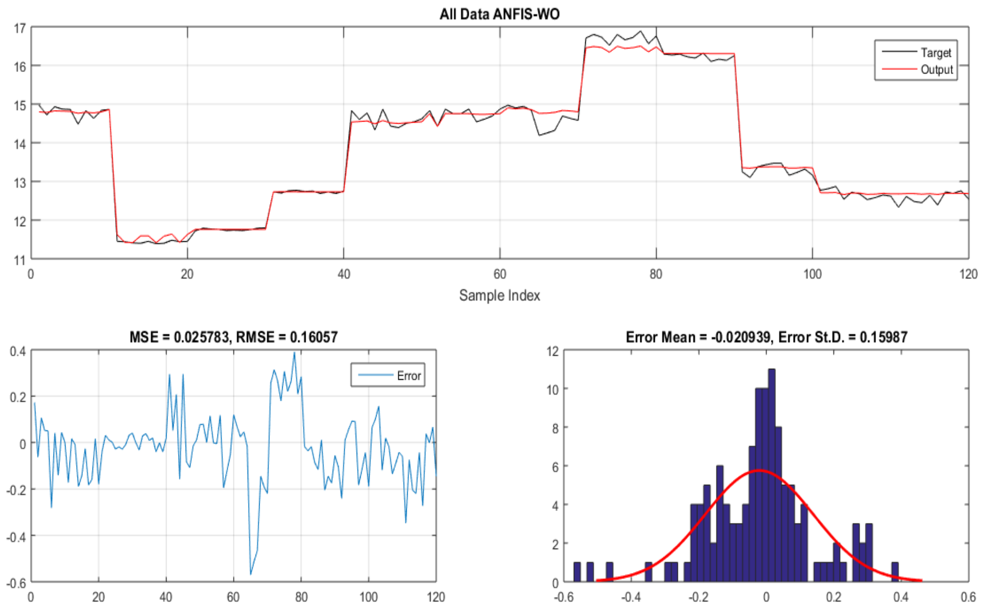

FIGURE S22. The target and predicted values of Methionine using 10fold cross-validation.

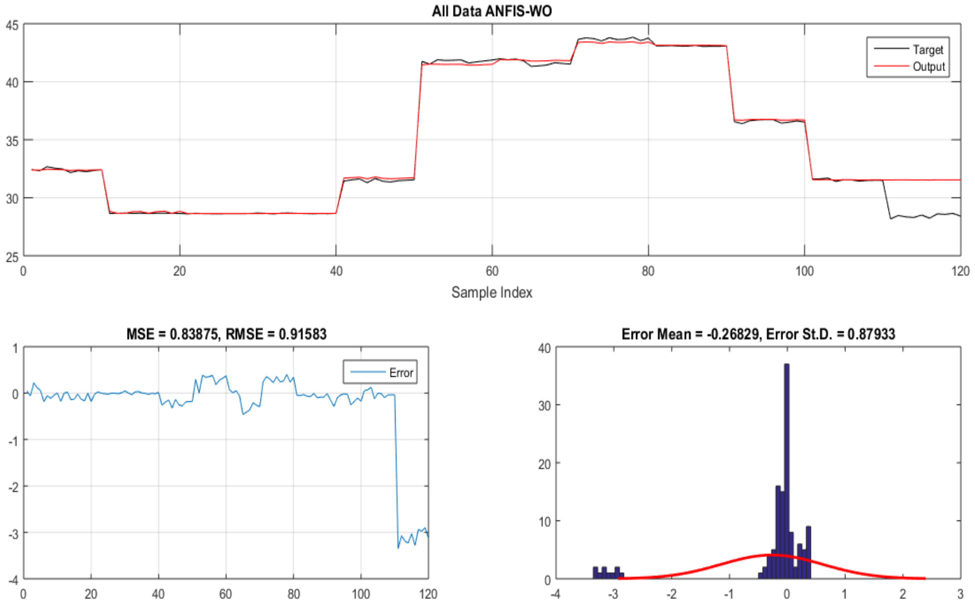

FIGURE S23. The target and predicted values of Proline using 10fold cross-validation.

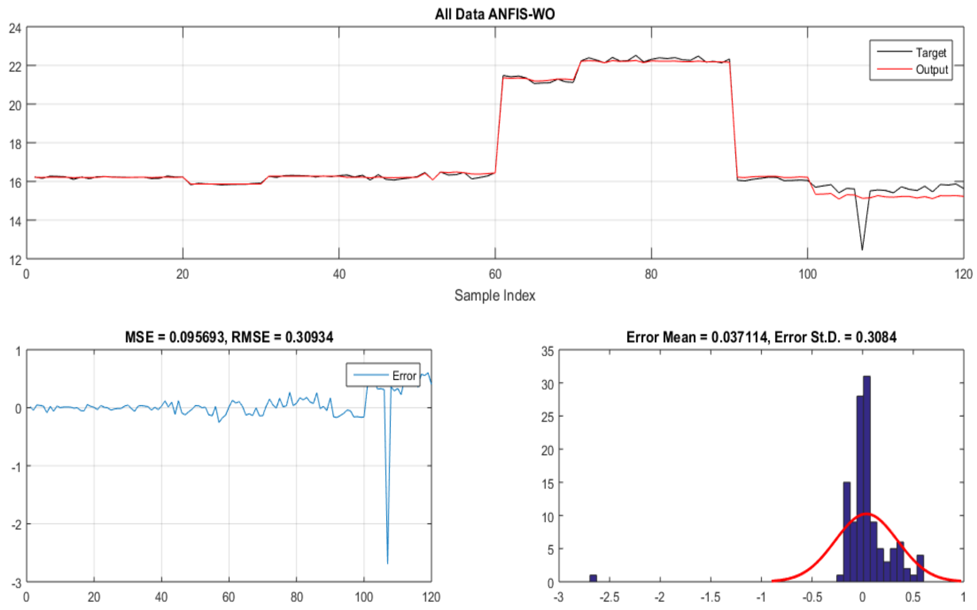

FIGURE S24. The target and predicted values of Isoleucine using 10fold cross-validation.

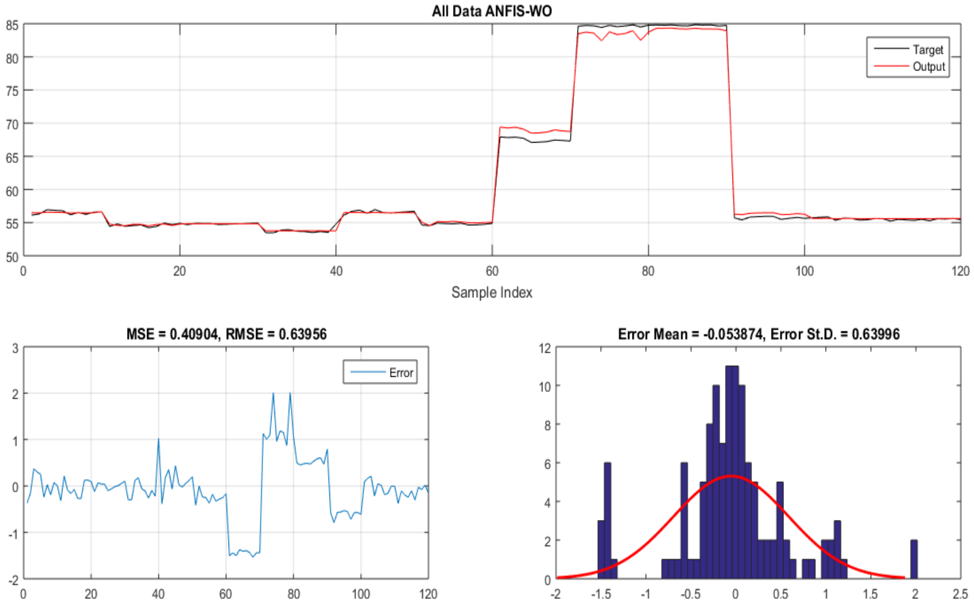

FIGURE S25. The target and predicted values of Glycine using 10fold cross-validation.

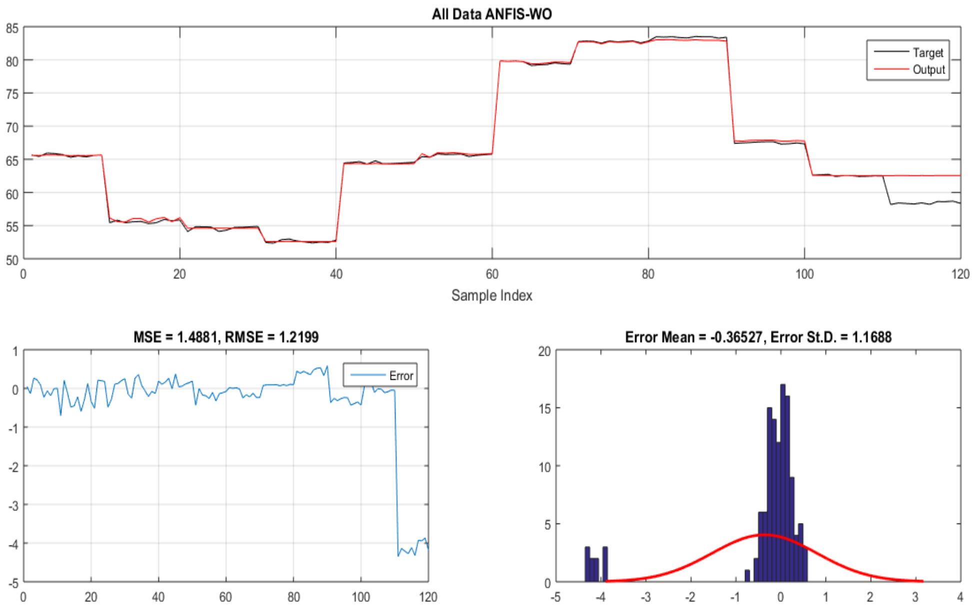

FIGURE S26. The target and predicted values of Glutamic using 10fold cross-validation.

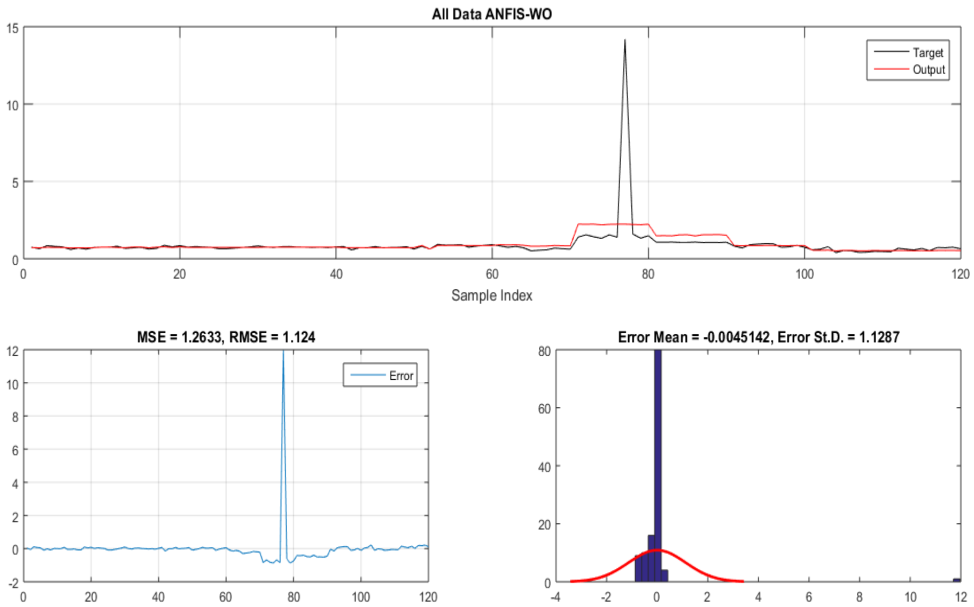

FIGURE S27. The target and predicted values of Cysteine using 10fold cross-validation.

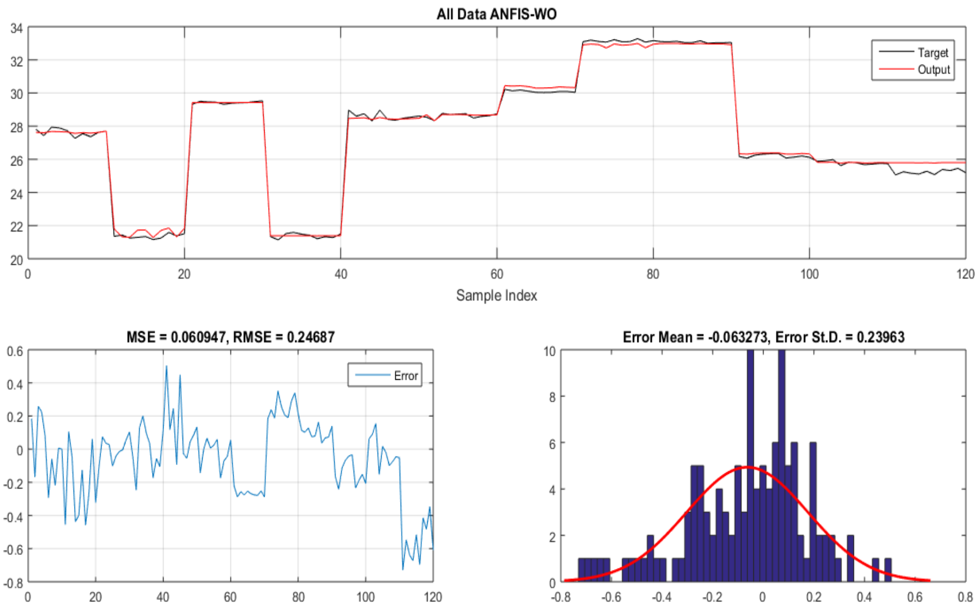

Figure S28. The target and predicted values of Arginine using 10fold cross-validation.

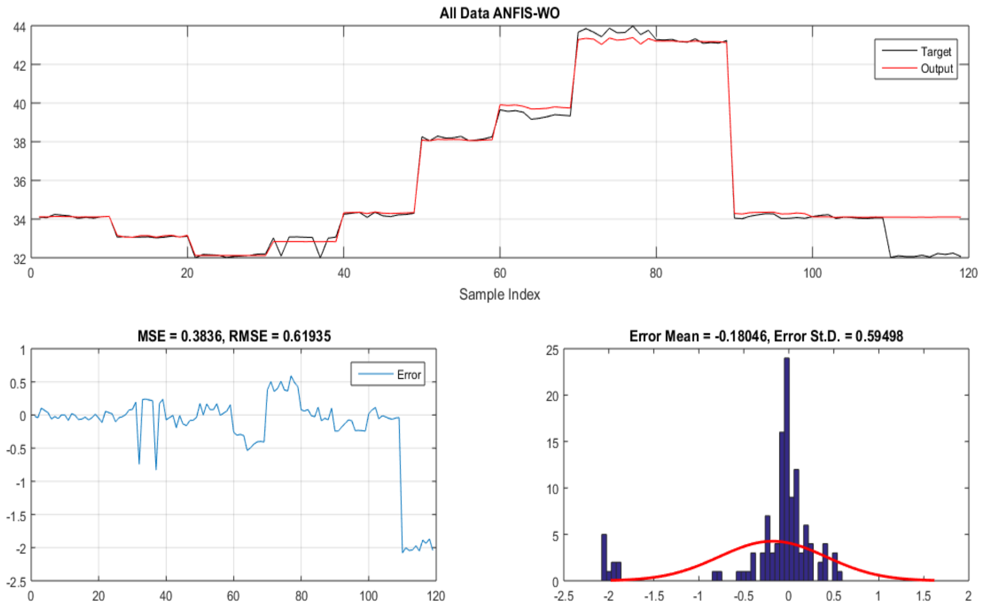

FIGURE S29. The target and predicted values of Leucine using 10fold cross-validation.

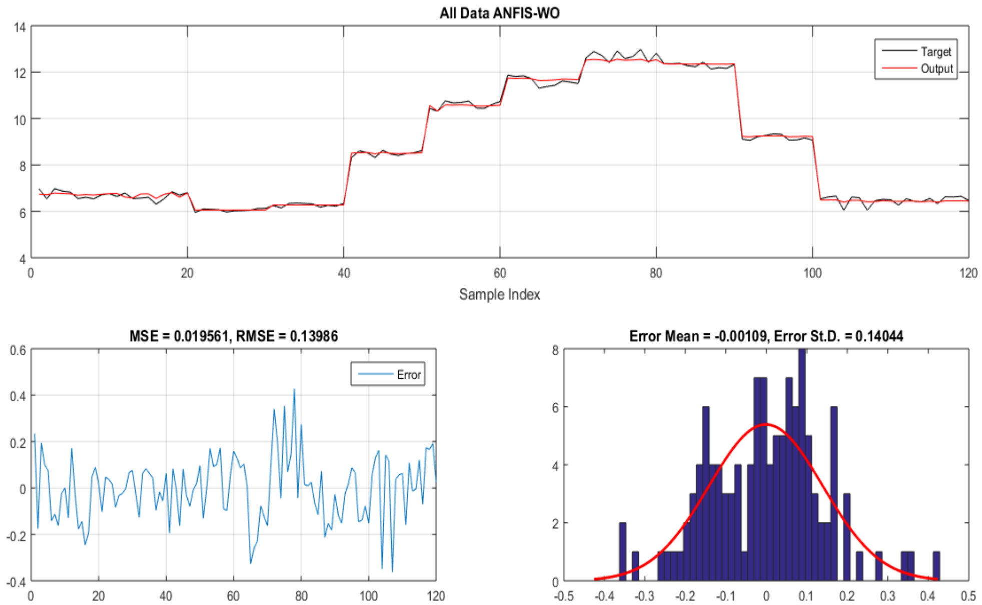

Figure S30. The target and predicted values of Histidine using 10fold cross-validation.
